# Supplementary material for: Year-Round Presence of Microcystins and Toxin-Producing Microcystis in the Water Column and Ice Cover of a Eutrophic Lake Located in the Continuous Permafrost Zone (Yakutia, Russia)
Source: Toxins (Basel). 2023 Jul 20;15(7):467. doi: 10.3390/toxins15070467 (PMC10467126; doi:10.3390/toxins15070467)
Supplement: Supplementary file 1 [file toxins-15-00467-s001.zip › toxins-2494445-supplementary.pdf]

Supplementary: Year-Round Presence of Microcystins and Toxin-Producing *Microcystis* in the Water Column and Ice Cover of a Eutrophic Lake Located in the Continuous Permafrost Zone (Yakutia, Russia)

Viktor A. Gabyshev [1], Sergey I. Sidelev [2,3], Ekaterina N. Chernova [4], Anna A. Vilnet [5], Denis A. Davydov [5,6], Sophia Barinova [7,*], Olga I. Gabysheva [1], Zoya A. Zhakovskaya [4] and Ivan V. Voronov [1]

Table S1a. Abundance (mln cells L^{-1}) and number of cyanobacterial species in surface water layer samples collected in Ytyk-Kyuyol Lake in ice-free period. " – " denotes to Species was not discovered in the sample, date format is DD.MM.YY

**Sampling Date**	**01.08.21**	**27.05.22**	**22.06.22**	**28.07.22**	**11.08.22**	**27.08.22**	**26.09.22**
Anabaena aequalis O.Borge	0.36	-	-	-	-	-	-
Anabaena augstumalis Schmidle	-	-	3.13	1.17	0.90	-	-
Anabaena laxa A.Braun	-	-	-	-	-	-	-
Anathece clathrata (West & G.S.West) Komárek, Kastovsky & Jezberová	31.01	0.70	3.58	-	-	2.25	1.05
Anathece endophytica (W. & G.S.West) Komárek, Kastovsky & Jezberová	-	-	-	-	0.27	0.19	0.10
Aphanizomenon flos-aquae Ralfs ex Bornet & Flahault	6.52	1.24	5.80	4.21	14.32	7.70	2.02
Aphanocapsa delicatissima West & G.S.West	-	-	2.30	1.83	-	1.44	0.93
Aphanocapsa grevillei (Berkeley) Rabenhorst	-	0.19	0.94	0.83	-	2.10	-
Aphanocapsa holsatica (Lemmermann) G.Cronberg & Komárek	-	-	-	-	-	-	-
Aphanocapsa planctonica (G.M.Smith) Komárek & Anagnostidis	4.37	-	-	-	-	-	-
Aphanothece microscopica Nägeli	-	-	-	-	-	0.01	-
Coelomoron pusillum (Van Goor) Komárek	-	-	-	-	-	-	-
Dolichospermum circinale (Rabenhorst ex Bornet & Flahault) Wacklin, Hoffmann & Komárek	-	-	-	0.19	-	-	-
Dolichospermum smithii (Komárek) Wacklin, L.Hoffmann & Komárek	-	-	-	0.28	-	-	-
Dolichospermum spiroides (Klebhan) Wacklin, L.Hoffmann & Komárek	0.65	-	-	-	-	-	-
Dolichospermum viguieri (Denis & Frémy) Wacklin, L.Hoffmann & Komárek	2.00	-	-	-	-	-	-
Gomphosphaeria aponina Kützing	-	-	-	-	-	-	-
Kamptonema chlorinum (Kützing ex Gomont) Strunecký, Komárek & J.Smarda	-	-	-	-	-	-	-
Merismopedia minima G.Beck in G.Beck & Zahlbruckner	198.68	-	-	-	-	-	-
Merismopedia tenuissima Lemmermann	-	0.37	1.14	0.97	1.38	0.89	-
Microcystis aeruginosa (Kützing) Kützing	-	2.07	52.94	35.55	43.58	33.32	16.46
Microcystis botrys Teiling	-	0.04	-	0.25	0.27	0.21	0.09
Microcystis flos-aquae (Wittrock) Kirchner	79.65	15.80	24.39	29.99	41.04	29.84	13.68
Microcystis novacekii (Komárek) Compère	-	-	-	-	-	-	-
Microcystis pulverea (H.C.Wood) Forti	13.33	-	-	-	-	-	-
Microcystis smithii Komárek & Anagnostidis	-	-	-	-	-	-	-
Microcystis wesenbergii (Komárek) Komárek ex Komárek	-	-	0.66	0.95	1.70	1.85	0.70
Oscillatoria limosa C.Agardh ex Gomont	-	0.01	-	-	-	-	-
Oscillatoria simplicissima Gomont	-	-	-	-	-	-	-
Phormidium ambiguum Gomont	-	-	0.24	-	-	-	-
Rhabdogloea smithii (Chodat & F.Chodat) Komárek	-	-	-	-	-	-	0.72
Snowella lacustris (Chodat) Komárek & Hindák	-	-	-	-	-	-	-
Woronichinia naegeliana (Unger) Elenkin	6.06	0.17	1.68	4.59	7.13	2.79	1.35
Number of discovered species in the sample	10	9	11	12	9	12	10

Table S1b. Abundance (mln cells L^{-1}) and number of cyanobacterial species in surface water layer samples collected in Ytyk-Kyuyol Lake in ice cover period. " – " denotes to Species was not discovered in the sample, date format is DD.MM.YY

**Sampling Date**	**04.10.21**	**10.12.21**	**05.01.22**	**16.02.22**	**24.03.22**	**22.04.22**	**24.10.22**	**25.11.22**
Anabaena aequalis O.Borge	-	-	-	-	-	-	-	-
Anabaena augstumalis Schmidle	-	-	-	-	-	-	-	-
Anabaena laxa A.Braun	-	-	-	-	-	-	-	-
Anathece clathrata (West & G.S.West) Komárek, Kastovsky & Jezberová	0.2527	-	-	0.0018	-	-	-	-
Anathece endophytica (W. & G.S.West) Komárek, Kastovsky & Jezberová	-	-	-	-	-	-	-	-
Aphanizomenon flos-aquae Ralfs ex Bornet & Flahault	0.6210	-	-	-	-	-	1.1884	-
Aphanocapsa delicatissima West & G.S.West	-	-	-	-	-	-	0.5473	-
Aphanocapsa grevillei (Berkeley) Rabenhorst	-	0.0020	-	0.0025	-	-	0.5691	0.0080
Aphanocapsa holsatica (Lemmermann) G.Cronberg & Komárek	-	-	-	-	-	-	-	-
Aphanocapsa planctonica (G.M.Smith) Komárek & Anagnostidis	-	-	-	-	-	-	-	-
Aphanothece microscopica Nägeli	-	-	-	-	0.0014	-	-	-
Coelomoron pusillum (Van Goor) Komárek	-	-	-	-	-	-	-	-
Dolichospermum circinale (Rabenhorst ex Bornet & Flahault) Wacklin, Hoffmann & Komárek	-	-	-	-	-	-	-	-
Dolichospermum smithii (Komárek) Wacklin, L.Hoffmann & Komárek	-	-	-	-	-	-	-	-
Dolichospermum spiroides (Klebhan) Wacklin, L.Hoffmann & Komárek	0.2470	-	-	-	-	-	-	-
Dolichospermum viguieri (Denis & Frémy) Wacklin, L.Hoffmann & Komárek	-	-	-	-	-	-	-	-
Gomphosphaeria aponina Kützing	-	-	-	-	-	-	-	-
Kamptonema chlorinum (Kützing ex Gomont) Strunecký, Komárek & J.Smarda	-	-	-	-	-	-	-	-
Merismopedia minima G.Beck in G.Beck & Zahlbruckner	0.1800	-	-	-	-	-	-	-
Merismopedia tenuissima Lemmermann	-	-	-	-	-	-	-	0.0008
Microcystis aeruginosa (Kützing) Kützing	13.2126	0.0110	-	-	-	-	8.4357	0.0180
Microcystis botrys Teiling	-	-	-	-	-	-	5.0748	-
Microcystis flos-aquae (Wittrock) Kirchner	11.2552	0.0090	0.0085	0.0082	0.0067	0.0019	7.9579	0.0160
Microcystis novacekii (Komárek) Compère	-	-	-	-	-	-	-	-
Microcystis pulverea (H.C.Wood) Forti	-	-	-	-	-	-	-	-
Microcystis smithii Komárek & Anagnostidis	-	-	-	-	-	-	-	-
Microcystis wesenbergii (Komárek) Komárek ex Komárek	-	-	-	-	-	-	0.3630	-
Oscillatoria limosa C.Agardh ex Gomont	-	-	-	-	-	-	-	-
Oscillatoria simplicissima Gomont	-	-	-	-	-	-	-	-
Phormidium ambiguum Gomont	-	-	-	-	-	-	-	-
Rhabdogloea smithii (Chodat & F.Chodat) Komárek	-	-	-	-	-	-	-	-
Snowella lacustris (Chodat) Komárek & Hindák	-	-	-	-	0.0028	-	-	-
Woronichinia naegeliana (Unger) Elenkin	0.6270	-	-	-	0.0024	-	-	-
Number of discovered species in the sample	7	3	1	3	4	1	7	4

Table S1c. Abundance (mln cells L^{-1}) and number of cyanobacterial species in bottom water layer samples collected in Ytyk-Kyuyol Lake in ice-free period. " – " denotes to Species was not discovered in the sample, date format is DD.MM.YY

**Sampling Date**	**22.06.22**	**28.07.22**	**27.08.22**	**26.09.22**
Anabaena aequalis O.Borge	-	-	-	-
Anabaena augstumalis Schmidle	1.55	-	-	-
Anabaena laxa A.Braun	-	-	-	0.33
Anathece clathrata (West & G.S.West) Komárek, Kastovsky & Jezberová	2.12	2.51	2.29	0.98
Anathece endophytica (W. & G.S.West) Komárek, Kastovsky & Jezberová	-	-	0.22	0.09
Aphanizomenon flos-aquae Ralfs ex Bornet & Flahault	2.86	5.14	6.25	-
Aphanocapsa delicatissima West & G.S.West	-	1.81	1.73	-
Aphanocapsa grevillei (Berkeley) Rabenhorst	-	1.01	-	0.75
Aphanocapsa holsatica (Lemmermann) G.Cronberg & Komárek	-	-	0.80	-
Aphanocapsa planctonica (G.M.Smith) Komárek & Anagnostidis	-	-	-	-
Aphanothece microscopica Nägeli	-	-	-	-
Coelomoron pusillum (Van Goor) Komárek	-	-	-	-
Dolichospermum circinale (Rabenhorst ex Bornet & Flahault) Wacklin, Hoffmann & Komárek	0.14	-	-	-
Dolichospermum smithii (Komárek) Wacklin, L.Hoffmann & Komárek	0.28	-	-	-
Dolichospermum spiroides (Klebhan) Wacklin, L.Hoffmann & Komárek	-	0.63	-	-
Dolichospermum viguieri (Denis & Frémy) Wacklin, L.Hoffmann & Komárek	-	-	-	-
Gomphosphaeria aponina Kützing	-	-	-	-
Kamptonema chlorinum (Kützing ex Gomont) Strunecký, Komárek & J.Smarda	-	-	-	0.22
Merismopedia minima G.Beck in G.Beck & Zahlbruckner	-	-	-	-
Merismopedia tenuissima Lemmermann	-	-	0.90	0.34
Microcystis aeruginosa (Kützing) Kützing	31.96	38.06	38.78	15.67
Microcystis botrys Teiling	0.17	0.23	-	-
Microcystis flos-aquae (Wittrock) Kirchner	17.99	28.19	30.86	12.33
Microcystis novacekii (Komárek) Compère	-	1.13	-	-
Microcystis pulverea (H.C.Wood) Forti	-	-	-	-
Microcystis smithii Komárek & Anagnostidis	-	-	-	-
Microcystis wesenbergii (Komárek) Komárek ex Komárek	0.64	-	1.18	0.62
Oscillatoria limosa C.Agardh ex Gomont	-	-	-	-
Oscillatoria simplicissima Gomont	-	-	-	-
Phormidium ambiguum Gomont	-	-	-	-
Rhabdogloea smithii (Chodat & F.Chodat) Komárek	-	-	-	-
Snowella lacustris (Chodat) Komárek & Hindák	-	-	-	0.03
Woronichinia naegeliana (Unger) Elenkin	2.95	3.24	2.60	1.30
Number of discovered species in the sample	10	10	10	11

Table S1d. Abundance (mln cells L^{-1}) and number of cyanobacterial species in bottom water layer samples collected in Ytyk-Kyuyol Lake in ice cover period. " – " denotes to Species was not discovered in the sample, date format is DD.MM.YY

**Sampling Date**	**10.12.21**	**05.01.22**	**16.02.22**	**24.03.22**	**22.04.22**	**24.10.22**	**25.11.22**
Anabaena aequalis O.Borge	-	-	-	-	-	-	-
Anabaena augstumalis Schmidle	-	-	-	-	-	-	-
Anabaena laxa A.Braun	-	-	-	-	-	-	-
Anathece clathrata (West & G.S.West) Komárek, Kastovsky & Jezberová	-	-	-	-	-	0.416	-
Anathece endophytica (W. & G.S.West) Komárek, Kastovsky & Jezberová	-	-	-	-	-	0.046	-
Aphanizomenon flos-aquae Ralfs ex Bornet & Flahault	-	-	-	-	-	0.594	-
Aphanocapsa delicatissima West & G.S.West	-	-	0.001	-	-	0.348	-
Aphanocapsa grevillei (Berkeley) Rabenhorst	0.003	-	-	0.002	-	-	0.010
Aphanocapsa holsatica (Lemmermann) G.Cronberg & Komárek	-	0.002	-	-	-	-	-
Aphanocapsa planctonica (G.M.Smith) Komárek & Anagnostidis	-	-	-	-	-	-	-
Aphanothece microscopica Nägeli	-	-	0.002	-	-	-	-
Coelomoron pusillum (Van Goor) Komárek	-	-	-	-	-	-	-
Dolichospermum circinale (Rabenhorst ex Bornet & Flahault) Wacklin, Hoffmann & Komárek	-	-	-	-	-	-	-
Dolichospermum smithii (Komárek) Wacklin, L.Hoffmann & Komárek	-	-	-	-	-	-	-
Dolichospermum spiroides (Klebhan) Wacklin, L.Hoffmann & Komárek	-	-	-	-	-	-	-
Dolichospermum viguieri (Denis & Frémy) Wacklin, L.Hoffmann & Komárek	-	-	-	-	-	-	-
Gomphosphaeria aponina Kützing	-	-	-	-	-	0.0001	-
Kamptonema chlorinum (Kützing ex Gomont) Strunecký, Komárek & J.Smarda	-	-	-	-	-	-	-
Merismopedia minima G.Beck in G.Beck & Zahlbruckner	-	-	-	-	-	-	-
Merismopedia tenuissima Lemmermann	-	-	-	-	-	0.097	-
Microcystis aeruginosa (Kützing) Kützing	0.010	0.011	-	-	-	7.080	0.020
Microcystis botrys Teiling	-	-	-	-	-	-	-
Microcystis flos-aquae (Wittrock) Kirchner	0.008	0.009	0.009	0.007	0.002	7.546	0.018
Microcystis novacekii (Komárek) Compère	-	-	-	-	-	-	-
Microcystis pulverea (H.C.Wood) Forti	-	-	-	-	-	-	-
Microcystis smithii Komárek & Anagnostidis	-	-	-	-	-	-	-
Microcystis wesenbergii (Komárek) Komárek ex Komárek	-	-	-	-	-	0.265	0.005
Oscillatoria limosa C.Agardh ex Gomont	-	-	-	-	-	-	-
Oscillatoria simplicissima Gomont	0.001	-	-	-	-	-	-
Phormidium ambiguum Gomont	-	-	-	-	-	-	-
Rhabdogloea smithii (Chodat & F.Chodat) Komárek	-	-	-	-	-	-	-
Snowella lacustris (Chodat) Komárek & Hindák	-	-	-	-	-	-	-
Woronichinia naegeliana (Unger) Elenkin	-	-	-	0.002	-	0.535	0.003
Number of discovered species in the sample	4	3	3	3	1	10	5

Table S1e. Abundance (mln cells L^{-1}) and number of cyanobacterial species in ice samples collected in Ytyk-Kyuyol Lake. " – " denotes to Species was not discovered in the sample, date format is DD.MM.YY

Sampling Date	10.12.21	05.01.22	16.02.22	24.03.22	22.04.22	24.10.22	25.11.22
Anabaena aequalis O.Borge	-	-	-	-	-	-	-
Anabaena augstumalis Schmidle	-	-	-	-	-	-	-
Anabaena laxa A.Braun	-	-	-	-	-	-	-
Anathece clathrata (West & G.S.West) Komárek, Kastovsky & Jezberová	-	-	-	-	-	-	-
Anathece endophytica (W. & G.S.West) Komárek, Kastovsky & Jezberová	-	-	-	-	-	-	-
Aphanizomenon flos-aquae Ralfs ex Bornet & Flahault	-	-	-	-	-	-	-
Aphanocapsa delicatissima West & G.S.West	-	-	-	-	-	-	-
Aphanocapsa grevillei (Berkeley) Rabenhorst	0.002	0.002	0.002	0.004	0.002	0.610	0.023
Aphanocapsa holsatica (Lemmermann) G.Cronberg & Komárek	-	-	-	-	-	-	-
Aphanocapsa planctonica (G.M.Smith) Komárek & Anagnostidis	-	-	-	-	-	-	-
Aphanothece microscopica Nägeli	-	-	-	-	0.001	-	-
Coelomoron pusillum (Van Goor) Komárek	0.001	-	-	0.003	0.001	-	-
Dolichospermum circinale (Rabenhorst ex Bornet & Flahault) Wacklin, Hoffmann & Komárek	-	-	-	-	-	-	-
Dolichospermum smithii (Komárek) Wacklin, L.Hoffmann & Komárek	-	-	-	-	-	-	-
Dolichospermum spiroides (Klebhan) Wacklin, L.Hoffmann & Komárek	-	-	-	-	-	-	-
Dolichospermum viguieri (Denis & Frémy) Wacklin, L.Hoffmann & Komárek	-	-	-	-	-	-	-
Gomphosphaeria aponina Kützing	-	-	-	-	-	-	-
Kamptonema chlorinum (Kützing ex Gomont) Strunecký, Komárek & J.Smarda	-	-	-	-	-	-	-
Merismopedia minima G.Beck in G.Beck & Zahlbruckner	-	-	-	-	-	-	-
Merismopedia tenuissima Lemmermann	-	-	-	-	-	-	-
Microcystis aeruginosa (Kützing) Kützing	-	0.010	-	0.002	0.002	16.527	0.032
Microcystis botrys Teiling	-	-	-	-	-	0.074	-
Microcystis flos-aquae (Wittrock) Kirchner	0.009	0.008	0.008	0.003	0.002	18.277	0.025
Microcystis novacekii (Komárek) Compère	-	-	-	-	-	-	-
Microcystis pulverea (H.C.Wood) Forti	-	-	-	-	-	-	-
Microcystis smithii Komárek & Anagnostidis	-	0.004	-	-	-	-	-
Microcystis wesenbergii (Komárek) Komárek ex Komárek	0.004	-	-	-	-	0.346	0.010
Oscillatoria limosa C.Agardh ex Gomont	-	-	-	-	-	-	-
Oscillatoria simplicissima Gomont	-	-	-	-	-	-	-
Phormidium ambiguum Gomont	-	-	-	-	-	-	-
Rhabdogloea smithii (Chodat & F.Chodat) Komárek	-	-	-	-	-	-	0.002
Snowella lacustris (Chodat) Komárek & Hindák	0.004	0.003	-	-	0.001	-	-
Woronichinia naegeliana (Unger) Elenkin	-	-	0.003	0.002	0.002	-	-
Number of discovered species in the sample	5	5	3	5	7	5	5

Table S2a. Biomass (mg L^{-1}) of cyanobacterial species in surface water layer samples collected in Ytyk-Kyuyol Lake in ice-free period. " – " denotes to Species was not discovered in the sample, date format is DD.MM.YY.

Sampling Date	01.08.21	27.05.22	22.06.22	28.07.22	11.08.22	27.08.22	26.09.22
Anabaena aequalis O.Borge	0.04	-	-	-	-	-	-
Anabaena augstumalis Schmidle	-	-	0.328	0.123	0.095	-	-
Anabaena laxa A.Braun	-	-	-	-	-	-	-
Anathece clathrata (West & G.S.West) Komárek, Kastovsky & Jezberová	0.04	0.001	0.004	-	-	0.003	0.001
Anathece endophytica (W. & G.S.West) Komárek, Kastovsky & Jezberová	-	-	-	-	0.0004	0.0003	0.0002
Aphanizomenon flos-aquae Ralfs ex Bornet & Flahault	0.60	0.018	0.082	0.060	0.203	0.109	0.029
Aphanocapsa delicatissima West & G.S.West	-	-	0.002	0.001	-	0.001	0.001
Aphanocapsa grevillei (Berkeley) Rabenhorst	-	0.008	0.037	0.033	-	0.083	-
Aphanocapsa holsatica (Lemmermann) G.Cronberg & Komárek	-	-	-	-	-	-	-
Aphanocapsa planctonica (G.M.Smith) Komárek & Anagnostidis	0.04	-	-	-	-	-	-
Aphanothece microscopica Nägeli	-	-	-	-	-	0.0002	-
Coelomoron pusillum (Van Goor) Komárek	-	-	-	-	-	-	-
Dolichospermum circinale (Rabenhorst ex Bornet & Flahault) Wacklin, Hoffmann & Komárek	-	-	-	0.060	-	-	-
Dolichospermum smithii (Komárek) Wacklin, L.Hoffmann & Komárek	-	-	-	0.025	-	-	-
Dolichospermum spiroides (Klebhan) Wacklin, L.Hoffmann & Komárek	0.15	-	-	-	-	-	-
Dolichospermum viguieri (Denis & Frémy) Wacklin, L.Hoffmann & Komárek	0.30	-	-	-	-	-	-
Gomphosphaeria aponina Kützing	-	-	-	-	-	-	-
Kamptonema chlorinum (Kützing ex Gomont) Strunecký, Komárek & J.Smarda	-	-	-	-	-	-	-
Merismopedia minima G.Beck in G.Beck & Zahlbruckner	0.03	-	-	-	-	-	-
Merismopedia tenuissima Lemmermann	-	0.001	0.004	0.003	0.005	0.003	-
Microcystis aeruginosa (Kützing) Kützing	-	0.096	2.460	1.652	2.025	1.549	0.765
Microcystis botrys Teiling	-	0.004	-	0.028	0.030	0.024	0.010
Microcystis flos-aquae (Wittrock) Kirchner	3.20	0.664	1.025	1.260	1.724	1.254	0.575
Microcystis novacekii (Komárek) Compère	-	-	-	-	-	-	-
Microcystis pulverea (H.C.Wood) Forti	0.20	-	-	-	-	-	-
Microcystis smithii Komárek & Anagnostidis	-	-	-	-	-	-	-
Microcystis wesenbergii (Komárek) Komárek ex Komárek	-	-	0.062	0.088	0.158	0.172	0.065
Oscillatoria limosa C.Agardh ex Gomont	-	0.002	-	-	-	-	-
Oscillatoria simplicissima Gomont	-	-	-	-	-	-	-
Phormidium ambiguum Gomont	-	-	0.033	-	-	-	-
Rhabdogloea smithii (Chodat & F.Chodat) Komárek	-	-	-	-	-	-	0.005
Snowella lacustris (Chodat) Komárek & Hindák	-	-	-	-	-	-	-
Woronichinia naegeliana (Unger) Elenkin	0.20	0.006	0.062	0.168	0.261	0.102	0.050

Table S2b. Biomass (mg L^{-1}) of cyanobacterial species in surface water layer samples collected in Ytyk-Kyuyol Lake in ice cover period. " – " denotes to Species was not discovered in the sample, date format is DD.MM.YY.

Sampling Date	04.10.21	10.12.21	05.01.22	16.02.22	24.03.22	22.04.22	24.10.22	25.11.22
Anabaena aequalis O.Borge	-	-	-	-	-	-	-	-
Anabaena augstumalis Schmidle	-	-	-	-	-	-	-	-
Anabaena laxa A.Braun	-	-	-	-	-	-	-	-
Anathece clathrata (West & G.S.West) Komárek, Kastovsky & Jezberová	0.0003	-	-	0.000001	-	-	-	-
Anathece endophytica (W. & G.S.West) Komárek, Kastovsky & Jezberová	-	-	-	-	-	-	-	-
Aphanizomenon flos-aquae Ralfs ex Bornet & Flahault	0.0111	-	-	-	-	-	0.0168	-
Aphanocapsa delicatissima West & G.S.West	-	-	-	-	-	-	0.0004	-
Aphanocapsa grevillei (Berkeley) Rabenhorst	-	0.0001	-	0.0001	-	-	0.0224	0.0003
Aphanocapsa holsatica (Lemmermann) G.Cronberg & Komárek	-	-	-	-	-	-	-	-
Aphanocapsa planctonica (G.M.Smith) Komárek & Anagnostidis	-	-	-	-	-	-	-	-
Aphanothece microscopica Nägeli	-	-	-	-	0.00003	-	-	-
Coelomoron pusillum (Van Goor) Komárek	-	-	-	-	-	-	-	-
Dolichospermum circinale (Rabenhorst ex Bornet & Flahault) Wacklin, Hoffmann & Komárek	-	-	-	-	-	-	-	-
Dolichospermum smithii (Komárek) Wacklin, L.Hoffmann & Komárek	-	-	-	-	-	-	-	-
Dolichospermum spiroides (Klebhan) Wacklin, L.Hoffmann & Komárek	0.0213	-	-	-	-	-	-	-
Dolichospermum viguieri (Denis & Frémy) Wacklin, L.Hoffmann & Komárek	-	-	-	-	-	-	-	-
Gomphosphaeria aponina Kützing	-	-	-	-	-	-	-	-
Kamptonema chlorinum (Kützing ex Gomont) Strunecký, Komárek & J.Smarda	-	-	-	-	-	-	-	-
Merismopedia minima G.Beck in G.Beck & Zahlbruckner	0.00003	-	-	-	-	-	-	-
Merismopedia tenuissima Lemmermann	-	-	-	-	-	-	-	0.000003
Microcystis aeruginosa (Kützing) Kützing	0.8730	0.0004	-	-	-	-	0.3920	0.0006
Microcystis botrys Teiling	-	-	-	-	-	-	0.5700	-
Microcystis flos-aquae (Wittrock) Kirchner	0.7297	0.0003	0.0003	0.0003	0.0001	0.0001	0.3344	0.0005
Microcystis novacekii (Komárek) Compère	-	-	-	-	-	-	-	-
Microcystis pulverea (H.C.Wood) Forti	-	-	-	-	-	-	-	-
Microcystis smithii Komárek & Anagnostidis	-	-	-	-	-	-	-	-
Microcystis wesenbergii (Komárek) Komárek ex Komárek	-	-	-	-	-	-	0.0336	-
Oscillatoria limosa C.Agardh ex Gomont	-	-	-	-	-	-	-	-
Oscillatoria simplicissima Gomont	-	-	-	-	-	-	-	-
Phormidium ambiguum Gomont	-	-	-	-	-	-	-	-
Rhabdogloea smithii (Chodat & F.Chodat) Komárek	-	-	-	-	-	-	-	-
Snowella lacustris (Chodat) Komárek & Hindák	-	-	-	-	0.0001	-	-	-
Woronichinia naegeliana (Unger) Elenkin	0.0447	-	-	-	0.0001	-	-	-

Table S2c. Biomass (mg L^{-1}) of cyanobacterial species in bottom water layer samples collected in Ytyk-Kyuyol Lake in ice-free period. " – " denotes to Species was not discovered in the sample, date format is DD.MM.YY.

**Sampling Date**	**22.06.22**	**28.07.22**	**27.08.22**	**26.09.22**
Anabaena aequalis O.Borge	-	-	-	-
Anabaena augstumalis Schmidle	0.162	-	-	-
Anabaena laxa A.Braun	-	-	-	0.013
Anathece clathrata (West & G.S.West) Komárek, Kastovsky & Jezberová	0.002	0.003	0.003	0.001
Anathece endophytica (W. & G.S.West) Komárek, Kastovsky & Jezberová	-	-	0.0003	0.0001
Aphanizomenon flos-aquae Ralfs ex Bornet & Flahault	0.041	0.073	0.088	-
Aphanocapsa delicatissima West & G.S.West	-	0.001	0.001	-
Aphanocapsa grevillei (Berkeley) Rabenhorst	-	0.040	-	0.029
Aphanocapsa holsatica (Lemmermann) G.Cronberg & Komárek	-	-	0.0003	-
Aphanocapsa planctonica (G.M.Smith) Komárek & Anagnostidis	-	-	-	-
Aphanothece microscopica Nägeli	-	-	-	-
Coelomoron pusillum (Van Goor) Komárek	-	-	-	-
Dolichospermum circinale (Rabenhorst ex Bornet & Flahault) Wacklin, Hoffmann & Komárek	0.045	-	-	-
Dolichospermum smithii (Komárek) Wacklin, L.Hoffmann & Komárek	0.024	-	-	-
Dolichospermum spiroides (Klebhan) Wacklin, L.Hoffmann & Komárek	-	0.050	-	-
Dolichospermum viguieri (Denis & Frémy) Wacklin, L.Hoffmann & Komárek	-	-	-	-
Gomphosphaeria aponina Kützing	-	-	-	-
Kamptonema chlorinum (Kützing ex Gomont) Strunecký, Komárek & J.Smarda	-	-	-	0.008
Merismopedia minima G.Beck in G.Beck & Zahlbruckner	-	-	-	-
Merismopedia tenuissima Lemmermann	-	-	0.003	0.001
Microcystis aeruginosa (Kützing) Kützing	1.485	1.769	1.802	0.728
Microcystis botrys Teiling	0.019	0.025	-	-
Microcystis flos-aquae (Wittrock) Kirchner	0.756	1.184	1.297	0.518
Microcystis novacekii (Komárek) Compère	-	0.040	-	-
Microcystis pulverea (H.C.Wood) Forti	-	-	-	-
Microcystis smithii Komárek & Anagnostidis	-	-	-	-
Microcystis wesenbergii (Komárek) Komárek ex Komárek	0.059	-	0.109	0.057
Oscillatoria limosa C.Agardh ex Gomont	-	-	-	-
Oscillatoria simplicissima Gomont	-	-	-	-
Phormidium ambiguum Gomont	-	-	-	-
Rhabdogloea smithii (Chodat & F.Chodat) Komárek	-	-	-	-
Snowella lacustris (Chodat) Komárek & Hindák	-	-	-	0.001
Woronichinia naegeliana (Unger) Elenkin	0.108	0.119	0.095	0.048

Table S2d. Biomass (mg L-1) of cyanobacterial species in bottom water layer samples collected in Ytyk-Kyuyol Lake in ice cover period. " – " denotes to Species was not discovered in the sample, date format is DD.MM.YY.

**Sampling Date**	**10.12.21**	**05.01.22**	**16.02.22**	**24.03.22**	**22.04.22**	**24.10.22**	**25.11.22**
Anabaena aequalis O.Borge	-	-	-	-	-	-	-
Anabaena augstumalis Schmidle	-	-	-	-	-	-	-
Anabaena laxa A.Braun	-	-	-	-	-	-	-
Anathece clathrata (West & G.S.West) Komárek, Kastovsky & Jezberová	-	-	-	-	-	0.0005	-
Anathece endophytica (W. & G.S.West) Komárek, Kastovsky & Jezberová	-	-	-	-	-	0.0001	-
Aphanizomenon flos-aquae Ralfs ex Bornet & Flahault	-	-	-	-	-	0.0084	-
Aphanocapsa delicatissima West & G.S.West	-	-	0.000001	-	-	0.0003	-
Aphanocapsa grevillei (Berkeley) Rabenhorst	0.0001	-	-	0.0001	-	-	0.0003
Aphanocapsa holsatica (Lemmermann) G.Cronberg & Komárek	-	0.000001	-	-	-	-	-
Aphanocapsa planctonica (G.M.Smith) Komárek & Anagnostidis	-	-	-	-	-	-	-
Aphanothece microscopica Nägeli	-	-	0.00004	-	-	-	-
Coelomoron pusillum (Van Goor) Komárek	-	-	-	-	-	-	-
Dolichospermum circinale (Rabenhorst ex Bornet & Flahault) Wacklin, Hoffmann & Komárek	-	-	-	-	-	-	-
Dolichospermum smithii (Komárek) Wacklin, L.Hoffmann & Komárek	-	-	-	-	-	-	-
Dolichospermum spiroides (Klebhan) Wacklin, L.Hoffmann & Komárek	-	-	-	-	-	-	-
Dolichospermum viguieri (Denis & Frémy) Wacklin, L.Hoffmann & Komárek	-	-	-	-	-	-	-
Gomphosphaeria aponina Kützing	-	-	-	-	-	0.00001	-
Kamptonema chlorinum (Kützing ex Gomont) Strunecký, Komárek & J.Smarda	-	-	-	-	-	-	-
Merismopedia minima G.Beck in G.Beck & Zahlbruckner	-	-	-	-	-	-	-
Merismopedia tenuissima Lemmermann	-	-	-	-	-	0.0003	-
Microcystis aeruginosa (Kützing) Kützing	0.0003	0.0003	-	-	-	0.3290	0.0007
Microcystis botrys Teiling	-	-	-	-	-	-	-
Microcystis flos-aquae (Wittrock) Kirchner	0.0003	0.0003	0.0003	0.0001	0.0001	0.3171	0.0006
Microcystis novacekii (Komárek) Compère	-	-	-	-	-	-	-
Microcystis pulverea (H.C.Wood) Forti	-	-	-	-	-	-	-
Microcystis smithii Komárek & Anagnostidis	-	-	-	-	-	-	-
Microcystis wesenbergii (Komárek) Komárek ex Komárek	-	-	-	-	-	0.0245	0.0006
Oscillatoria limosa C.Agardh ex Gomont	-	-	-	-	-	-	-
Oscillatoria simplicissima Gomont	0.0001	-	-	-	-	-	-
Phormidium ambiguum Gomont	-	-	-	-	-	-	-
Rhabdogloea smithii (Chodat & F.Chodat) Komárek	-	-	-	-	-	-	-
Snowella lacustris (Chodat) Komárek & Hindák	-	-	-	-	-	-	-
Woronichinia naegeliana (Unger) Elenkin	-	-	-	0.0001	-	0.0196	0.0001

Table S2e. Biomass (mg L-1) of cyanobacterial species in ice samples collected in Ytyk-Kyuyol Lake. "– " denotes to Species was not discovered in the sample, date format is DD.MM.YY.

**Sampling Date**	**10.12.21**	**05.01.22**	**16.02.22**	**24.03.22**	**22.04.22**	**24.10.22**	**25.11.22**
Anabaena aequalis O.Borge	-	-	-	-	-	-	-
Anabaena augstumalis Schmidle	-	-	-	-	-	-	-
Anabaena laxa A.Braun	-	-	-	-	-	-	-
Anathece clathrata (West & G.S.West) Komárek, Kastovsky & Jezberová	-	-	-	-	-	-	-
Anathece endophytica (W. & G.S.West) Komárek, Kastovsky & Jezberová	-	-	-	-	-	-	-
Aphanizomenon flos-aquae Ralfs ex Bornet & Flahault	-	-	-	-	-	-	-
Aphanocapsa delicatissima West & G.S.West	-	-	-	-	-	-	-
Aphanocapsa grevillei (Berkeley) Rabenhorst	0.00005	0.0001	0.0001	0.0001	0.00005	0.0240	0.0008
Aphanocapsa holsatica (Lemmermann) G.Cronberg & Komárek	-	-	-	-	-	-	-
Aphanocapsa planctonica (G.M.Smith) Komárek & Anagnostidis	-	-	-	-	-	-	-
Aphanothece microscopica Nägeli	-	-	-	-	0.00002	-	-
Coelomoron pusillum (Van Goor) Komárek	0.00003	-	-	0.0001	0.00002	-	-
Dolichospermum circinale (Rabenhorst ex Bornet & Flahault) Wacklin, Hoffmann & Komárek	-	-	-	-	-	-	-
Dolichospermum smithii (Komárek) Wacklin, L.Hoffmann & Komárek	-	-	-	-	-	-	-
Dolichospermum spiroides (Klebhan) Wacklin, L.Hoffmann & Komárek	-	-	-	-	-	-	-
Dolichospermum viguieri (Denis & Frémy) Wacklin, L.Hoffmann & Komárek	-	-	-	-	-	-	-
Gomphosphaeria aponina Kützing	-	-	-	-	-	-	-
Kamptonema chlorinum (Kützing ex Gomont) Strunecký, Komárek & J.Smarda	-	-	-	-	-	-	-
Merismopedia minima G.Beck in G.Beck & Zahlbruckner	-	-	-	-	-	-	-
Merismopedia tenuissima Lemmermann	-	-	-	-	-	-	-
Microcystis aeruginosa (Kützing) Kützing	-	0.0003	-	0.0001	0.0001	0.7680	0.0011
Microcystis botrys Teiling	-	-	-	-	-	0.0083	-
Microcystis flos-aquae (Wittrock) Kirchner	0.0003	0.0003	0.0003	0.0002	0.0001	0.7680	0.0008
Microcystis novacekii (Komárek) Compère	-	-	-	-	-	-	-
Microcystis pulverea (H.C.Wood) Forti	-	-	-	-	-	-	-
Microcystis smithii Komárek & Anagnostidis	-	0.0001	-	-	-	-	-
Microcystis wesenbergii (Komárek) Komárek ex Komárek	0.0004	-	-	-	-	0.0320	0.0011
Oscillatoria limosa C.Agardh ex Gomont	-	-	-	-	-	-	-
Oscillatoria simplicissima Gomont	-	-	-	-	-	-	-
Phormidium ambiguum Gomont	-	-	-	-	-	-	-
Rhabdogloea smithii (Chodat & F.Chodat) Komárek	-	-	-	-	-	-	0.00003
Snowella lacustris (Chodat) Komárek & Hindák	0.0002	0.0002	-	-	0.00004	-	-
Woronichinia naegeliana (Unger) Elenkin	-	-	0.0001	0.0001	0.0001	-	-

Table S3. Intracellular MCs congeners concentration (ng L^{-1}) detected in cells filtered from natural water and ice samples. Sample types: SW – surface water, BW – bottom water, I – ice sample. " – " denotes to the concentration below LOD (< 0,1 ng L^{-1}), date format is DD.MM.YY.

Sample type	Sampling date	[D-Asp^3] MC-LR	MC-LR	[D-Asp^3] MC-RR	MC-RR	MC-YR	[D-Asp^3] MC-YR	MC-WR	[D-Asp^3, ADMAdda^5] MC-LR	Total content
SW	01.08.21	16.0	--300.0	11.0	354.0	110.0	7.0	-	5.0	803.0
SW	04.10.21	4.2	--100.3	22.8	152.4	-	-	-	-	279.7
SW	10.12.21	-	4.1	-	6.9	-	-	-	-	11.0
SW	05.01.22	-	-	-	2.5	-	-	-	-	2.5
SW	16.02.22	-	-	-	0.8	-	-	-	-	0.8
SW	24.03.22	-	-	-	-	-	-	-	-	< 0.1
SW	22.04.22	-	-	-	-	-	-	-	-	< 0.1
SW	27.05.22	-	-	-	1.2	-	-	-	-	1.2
SW	22.06.22	-	-	0.4	16.3	-	-	-	-	16.6
SW	28.07.22	-	3.6	-	7.1	-	-	-	-	10.7
SW	11.08.22	1.8	66.5	-	57.0	9.1	-	1.1	-	135.5
SW	27.08.22	0.5	22.3	0.7	10.0	1.9	-	-	-	35.3
SW	26.09.22	0.2	5.5	-	4.2	0.5	-	-	-	10.3
SW	24.10.22	-	2.8	-	1.6	0.2	-	-	-	4.6
SW	25.11.22	-	1.0	-	1.3	-	-	-	-	2.3
BW	10.12.21	-	1.3	-	2.2	-	-	-	-	3.5
BW	05.01.22	-	-	-	2.4	-	-	-	-	2.4
BW	16.02.22	-	-	-	0.8	-	-	-	-	0.8
BW	24.03.22	-	-	-	-	-	-	-	-	< 0.1
BW	22.04.22	-	-	-	-	-	-	-	-	< 0.1
BW	22.06.22	-	38.1	-	-	-	-	-	-	38.1
BW	28.07.22	-	3.0	-	4.8	-	-	-	-	7.8
BW	27.08.22	0.8	44.0	-	18.0	3.2	-	0.8	-	66.8
BW	26.09.22	0.1	4.4	-	2.5	0.4	-	-	-	7.4
BW	24.10.22	0.1	2.9	-	1.9	0.6	-	-	-	5.5
BW	25.11.22	-	2.0	-	1.2	0.3	-	-	-	3.5
I	10.12.21	-	6.3	-	5.5	-	-	-	-	11.8
I	05.01.22	-	-	-	0.3	-	-	-	-	0.3
I	16.02.22	-	-	-	0.2	-	-	-	-	0.2
I	24.03.22	-	-	-	-	-	-	-	-	< 0.1
I	22.04.22	-	-	-	-	-	-	-	-	< 0.1
I	24.10.22	0.9	27.3	-	14.4	5.5	0.2	-	0.4	48.6
I	25.11.22	-	1.9	-	1.1	0.3	-	-	-	3.2

Table S4. Extracellular MCs congeners concentration detected in filtered surface water. " – " denotes to the concentration below LOD (< 0,1 ng L^{-1}), date format is DD.MM.YY.

Sampling date	MC-LR	[D-Asp³]MC-RR	MC-RR	Total content, ng L^{-1}
22.06.22	6.3	0.2	13.3	19.8
28.07.22	-	-	1.6	1.6
11.08.22	1.4	-	0.5	1.9

Table S5. Mean values of total abundance and biomass of cyanobacterial species and standard deviation

Sampling Date	Sampling medium	Depth of sampling layer	Mean value of total abundance, cells x $10^6 \cdot L^{-1}$ / Standard deviation	Mean value of total biomass, $mg \cdot L^{-1}$ / Standard deviation
01.08.2021	water	surface	342.63/6.5874	4.8/0.563
04.10.2021	water	surface	26.3955/1.1098	1.6802/0.0039
10.12.2021	water	bottom	0.0215/0.0012	0.0008/0.0001
10.12.2021	water	surface	0.022/0.0015	0.0007/0.0001
10.12.2021	ice	-	0.0191/0.0013	0.001/0.0001
05.01.2022	water	bottom	0.0212/0.0013	0.0006/0.0001
05.01.2022	water	surface	0.0085/0.0006	0.0003/0.0
05.01.2022	ice	-	0.027/0.0019	0.001/0.0001
16.02.2022	water	bottom	0.0113/0.0008	0.0003/0.0
16.02.2022	water	surface	0.0125/0.0008	0.0004/0.0
16.02.2022	ice	-	0.0122/0.0003	0.0004/0.0
24.03.2022	water	bottom	0.0107/0.0009	0.0002/0.0
24.03.2022	water	surface	0.0133/0.0002	0.0003/0.0
24.03.2022	ice	-	0.0143/0.0001	0.0006/0.0
22.04.2022	water	bottom	0.0017/0.0001	0.0001/0.0
22.04.2022	water	surface	0.0019/0.0002	0.0001/0.0
22.04.2022	ice	-	0.0102/0.0005	0.0004/0.0
27.05.2022	water	surface	20.5961/1.9237	0.8/0.0841
22.06.2022	water	surface	96.8001/5.4476	4.0973/0.3234
22.06.2022	water	bottom	60.6626/4.5519	2.7011/0.1769
28.07.2022	water	surface	80.7907/1.5661	3.4987/0.0947
28.07.2022	water	bottom	81.9378/2.9724	3.303/0.2188
11.08.2022	water	surface	110.5921/5.9092	4.5/0.1908
27.08.2022	water	surface	82.6061/3.5663	3.2987/0.2731
27.08.2022	water	bottom	85.6118/7.8648	3.3988/0.3784
26.09.2022	water	surface	37.0918/1.2062	1.4991/0.0698
26.09.2022	water	bottom	32.6578/1.6237	1.405/0.0694
24.10.2022	water	surface	24.1362/1.1517	1.3696/0.1759
24.10.2022	water	bottom	16.928/0.405	0.6997/0.082
24.10.2022	ice	-	35.8333/2.9251	1.6003/0.111
25.11.2022	water	surface	0.0428/0.0023	0.0014/0.0001
25.11.2022	water	bottom	0.056/0.0021	0.0022/0.0003
25.11.2022	ice	-	0.0924/0.0027	0.0038/0.0
